# Blended Care Intervention for Cancer Aftercare in General Practice Centers: Protocol for a Randomized Controlled Trial

**DOI:** 10.2196/64662

**Published:** 2025-02-12

**Authors:** Michelle J M Smits, Catherine A W Bolman, Ilse Mesters, Lilian Lechner

**Affiliations:** 1 Department of Health Psychology Faculty of Psychology Open University of the Netherlands Heerlen Netherlands; 2 Department of Epidemiology Faculty of Health, Medicine and Life Sciences Maastricht University Maastricht Netherlands

**Keywords:** cancer aftercare, general practice, blended care, eHealth, randomized controlled trial, cost effectiveness, general practitioners, online intervention

## Abstract

**Background:**

Combining effective eHealth programs with face-to-face consultations in general practice may help general practitioners care for survivors of cancer.

**Objective:**

This study protocol describes a 2-armed randomized controlled trial to evaluate the cost-effectiveness of a blended intervention integrating the Cancer Aftercare Guide in general practice centers (GPCs).

**Methods:**

A parallel-group design will compare an intervention group with a waiting list control group. Participants will be nested within GPCs and randomization will occur at the GPC level. The participants in the intervention group will receive a blended care intervention. In contrast, the participants in the waiting list control group will receive care as usual for the duration of this study and will receive the online intervention afterward. All participants will be asked to complete an online questionnaire at baseline, 6 months, and 12 months after baseline, measuring self-reported adherence to lifestyle recommendations, psychosocial well-being, and quality of life. A process evaluation and cost evaluation are also included in this study. The effects will be evaluated based on differences in residual change scores between intervention and control group participants, using multilevel linear regression analyses. Moreover, effect analyses will be supplemented with Bayes factor analyses. Finally, an economic evaluation will be conducted from a societal perspective and will include medical costs, productivity costs, and costs of the blended care intervention.

**Results:**

This study was funded in July 2020. Data collection started in August 2022 and is likely to be completed by April 2025. As of December 2024, a total of 127 participants have been included in this study, recruited across 26 GPCs in the Netherlands. Data analysis will commence once data collection is completed. Data analysis is estimated to start in the spring of 2025. The results will likely be published in 2026.

**Conclusions:**

The results will provide insight into the effectiveness of blended care and may be relevant to cancer aftercare, general practice, and the field of eHealth implementation in general. Potential challenges lie in recruitment due to the strain on the health care system since the COVID-19 pandemic.

**Trial Registration:**

ISRCTN ISRCTN12451453; https://www.isrctn.com/ISRCTN12451453

**International Registered Report Identifier (IRRID):**

DERR1-10.2196/64662

## Introduction

In the coming decades, the growing number of survivors of cancer will challenge health care worldwide. Global data show the increased incidence and survival rates, indicating an increase in the number of survivors of cancer worldwide [1]. Data for the Netherlands (total population of 17 million) predict that in 2032, a total of 1.4 million people will be receiving treatment for cancer or will have been successfully treated for cancer [2]. This underlines the urgency of studying the needs of survivors and finding ways to meet these needs to safeguard the quality of life (QoL) after disease and treatment.

After treatment, survivors of cancer may face physical, psychological, and psychosocial challenges that affect their transition to normal life. In addition, survivors of cancer must adhere to lifestyle recommendations regarding physical activity (PA), diet, smoking cessation, and alcohol consumption to prevent recurrence or the development of comorbidities [3]. In reality, however, many survivors of cancer find it difficult to adhere to these guidelines [4]. They seek support to help them recover and cope with the effects of their disease [5]. However, due to a shortage of health care professionals and increasing demand for care in the general population due to aging, survivors of cancer may not always receive the support they need.

Over the past decades, several eHealth interventions have been introduced to support survivors of cancer in their healthy recovery. The online Cancer Aftercare Guide (CAG) is an example of such an intervention [6,7]. The CAG is a web-based eHealth intervention that targets lifestyle and common psychological and psychosocial problems experienced by survivors by promoting self-management of these problems using evidence-based techniques, such as cognitive behavioral therapy and problem-solving therapy. Effectiveness evaluation has shown that the CAG is effective in reducing fatigue, depression, and anxiety and in increasing PA and healthy eating [8-11]. This shows that the CAG can help survivors cope with common cancer-related problems and can help reduce the risk of future disease by promoting a healthy lifestyle.

The use of eHealth for applications such as disease management and patient data sharing is encouraged [12,13], but in reality, effective eHealth interventions struggle to reach their target population [7,14,15]. For survivors of cancer in particular, it has been found that while they use the internet to search for health information, their use of eHealth self-help programs is not common [16]. One reason for this may be that many eHealth interventions are not implemented in everyday practice [17]. To improve the use of eHealth, the Dutch eHealth Monitor suggests that health care providers play an important role in informing patients about eHealth self-help programs and that health care workflows should be designed to facilitate the use of eHealth through blended care [18]. Similarly, in the case of the CAG, Willems et al [11] and Kanera [6] state that the online intervention could be offered in a blended approach, where patient-therapist interaction is provided, to promote patient engagement with the intervention.

Apart from a biyearly follow-up aimed at detecting recurrence, no structural care is currently provided for survivors of cancer in the Netherlands. For issues related to cancer survivorship, patients are referred to their general practitioner (GP) [19], resulting in on-demand care requests. In addition, GPs are responsible for tertiary prevention, which targets healthy lifestyle behaviors in survivors of cancer. With the increasing number of survivors of cancer, this demand will pose a significant challenge to general practice workflows. Therefore, a structured approach is essential for GPs to meet the needs of their patients. This is illustrated by the Dutch College of General Practitioners (in Dutch: *Nederlands Huisartsen Genootschap*), which stated that general practice could structurally provide cancer aftercare [20]. Implementing the CAG in general practice in a blended care setting could provide a solution to the growing demand for cancer aftercare by promoting healthy recovery and improving self-management of problems, ultimately reducing the care needs of survivors of cancer.

This study protocol outlines a randomized controlled trial (RCT) to investigate the cost-effectiveness and associated process evaluation of a blended care approach offering the CAG in general practice. The blended care intervention has been co-designed with GPs, practice nurses (PNs), and survivors in a separate study (MJM Smits, unpublished data, 2025) and has been tested in a pilot study before starting this RCT.

## Methods

### Study Design

An RCT will be conducted to compare the effectiveness of the blended CAG intervention between the intervention group and the waiting list control group. A general practice center (GPC) is usually run by at least 1 GP, supported by one or more medical assistants. In addition, many Dutch GPs are supported by PNs who are dedicated to patients with chronic disease, older adult care, and mental health care. The PNs provide additional care for identified health problems, such as asthma, chronic obstructive pulmonary disease, and diabetes (for the somatic nurse) or mental health problems (for the mental health nurse). The intervention protocol is implemented by either the GP or the PN (under the supervision of the GP). A full list of participating GPCs can be requested from the researchers.

### Recruitment of GPCs

GPCs will be recruited through a variety of channels, including calls published in online primary care newsletters distributed by Regional Collaborative Care Groups or special interest groups dedicated to oncology care or lifestyle counseling in primary care. In addition, direct mail will also be sent out by the research team. If no response is received within 4 weeks, the direct mailing will be followed by telephone calls to discuss potential participation. GPCs are eligible to participate if they are located in the Netherlands. If the GPC agrees to participate in this study, an appointment will be made for the researcher to visit the GPC to give instructions on this study protocol.

### Recruitment of Participants

#### Overview

Survivors of cancer will be recruited by general practice personnel (GPP: either GP, PN, or assistants). From the GP’s electronic medical record (in Dutch: *Huisartsen Informatie Systeem*), the GPP will select patients who meet the following inclusion criteria: (1) patients who have completed primary treatment of cancer (eg, radiotherapy, chemotherapy, or surgery), with the last treatment having been between 6 weeks and 3 years ago, or who belong to a watchful waiting condition (eg, option for prostate cancer patients); (2) patients who are 18 years of age or older; (3) patients who are able to read and speak Dutch; (4) patients without a serious medical, psychiatric, or cognitive condition that would interfere with participation; (5) patients who have access to the internet and at least minimal experience of using it; and (6) patients who have access to a computer or a tablet.

If desired, the selection of participants can be assisted by the researcher (under the supervision of the GP).

Eligible patients will be invited to participate in this study using an invitation package distributed by the GPC. The information package consists of an information letter, an informed consent form (Multimedia Appendix 1), and a prepaid return envelope. Survivors of cancer who agree to participate will need to sign the consent form and return it to the research team with the return envelope. The researchers will notify the GPC when the consent form has been received and stored. Enrolled patients will receive a welcome email from the researchers, informing them of their randomization (intervention or control group) and providing a link to the first online self-report questionnaire (baseline measurement). Awareness of randomization can lead to selection bias, but studies on survivors of cancer have shown that this effect is minimal [8-11]. Informing patients about the group to which they are assigned is required in the Netherlands by the Medical Research Involving Subjects Act.

#### The CAG Intervention

The online CAG program provides personalized information in 8 modules covering healthy lifestyles and common psychosocial issues related to cancer survivorship (ie, PA, diet, smoking cessation, alcohol use, fatigue, anxiety and depression, return to work, and social relationships). The CAG will use data from the baseline assessment to create the Module Referral Advice (MRA; Multimedia Appendix 2). The MRA presents personal outcomes across the 8 subtopics featured in the CAG and advises the participant to visit the module that corresponds to their greatest need, as indicated by the MRA. The modules consist of textual information and advice, video clips (in which former cancer patients share their survivorship experiences), and tasks or exercises that the participants can complete independently.

#### Intervention Procedure

Participants in the intervention group will receive the CAG blended care intervention. This means that after completing the baseline measurement, they will be given access to the online CAG. Participants will use the online CAG program independently at home. In addition, they will be invited to 2 consultations with their GP or PN. Within the online CAG, the intervention group participants will receive personalized advice on 8 topics related to cancer survivorship based on their results at baseline. This is represented by the MRA. Participants are asked to share their MRA results with the GP or PN at their first consultation. During the first consultation, the GP or PN and the participant will discuss the MRA results. Through shared decision-making, the GP or PN will motivate the participant to choose at least 1 of the 8 subtopics to focus on for the next 4 to 6 weeks, after which the follow-up consultation will take place. During the follow-up consultation, the GP or PN will enquire about the participant’s progress with the chosen topic or topics. Any questions or problems the participant may have in interpreting the (lifestyle) advice given by the program will be addressed in the personal consultation. After the follow-up consultation, the participant will retain access to the online CAG intervention for up to 6 months after the start of the baseline measurement.

After completing the baseline measurement, participants in the control group are directed to a message thanking them for completing the questionnaire, asking them to log out and return only for the next measurement. Per GP care, participants in the control group will receive care as usual, which is mostly initiated by patient request and is usually complaint-driven. At the end of this study, the researchers will actively inform and encourage control group participants to finally use the online CAG intervention. Study groups will have full access to other interventions during the trial, but only on their initiative. The use of cointervention will be assessed by questionnaires at all time points.

### Measurements

All participants will be asked to complete an online questionnaire at baseline, 6 months, and 12 months after baseline. GPs and PNs will not be present during the self-report questionnaire (participants will complete this assessment digitally at home). Therefore, no influence of the GP or PN on the Patient Reported Outcome Measures is expected. The questionnaires will measure self-reported lifestyle behaviors, behavioral determinants, and experienced psychological problems (see the *Outcomes* section).

The second questionnaire, administered 6 months after baseline, will also include items measuring health-related costs and, for intervention group participants only, a process evaluation questionnaire. In addition, biomedical measurements will be taken from all participants at 6 months after baseline. Blood pressure, total cholesterol/high-density lipoprotein cholesterol, and blood glucose were originally planned for the biomedical measurement. However, due to current circumstances in Dutch primary care (see the *Discussion* section), total cholesterol/high-density lipoprotein cholesterol and blood glucose measurements will be discontinued in consultation with the funding agency.

Nonresponse will be prevented by using previously effective protocols, including automated reminders 1 week and again 3 weeks after the distribution of questionnaires to patients [7,21] and email reminders to GPCs to ensure blood pressure measurement at 6 months. The quality control procedures ensure that there will be little nonresponse [8-11]. To improve the engagement of GPCs, relationships will be maintained through regular emails or phone calls to the designated contact person. In addition, a Christmas card will be sent each year on behalf of the research team. In addition, the GPCs will be offered support in patient selection and patient contact by a researcher on several occasions during this study.

### Outcomes

#### Overview

The primary outcomes of this study are changes in lifestyle behaviors, measured by validated self-report questionnaires. PA will be assessed using the self-report Short Questionnaire to Assess Health [22,23]. Smoking behavior will be assessed using a validated (self-report) abstinence scale [24,25]. Alcohol consumption will be measured using a standardized alcohol consumption scale [26]. Dietary behavior will be assessed using a food frequency questionnaire on saturated fat intake and fruit and vegetable consumption [27-29].

Secondary outcomes are changes in experienced psychosocial problems such as anxiety and depression, fatigue, health-related QoL, and health-related costs, which will be assessed using validated self-report questionnaires. Anxiety and depression will be assessed using the Hospital Anxiety and Depression Scale [30,31]. Fatigue is assessed using the Checklist Individual Strength [32,33]. Health-related QoL is measured using the abbreviated European Organisation for Research and Treatment of Cancer Quality of Life Questionnaire [34,35]. Health-related costs are considered from a societal perspective and are measured as health care consumption in the last 3 months using the Institute for Medical Technology Assessment Medical Consumption Questionnaire, productivity losses in the last 4 weeks for both paid and unpaid labor using the Institute for Medical Technology Assessment Productivity Cost Questionnaire, and quality-adjusted life years (QALY) using the EQ-5D-5L [25,36-42]. All questionnaires used are validated, we do not conduct additional validation research as part of this research project.

Secondary outcomes are also blood pressure measurements, taken from all participants at 6 months postbaseline. Blood pressure will be assessed by clinical measurements performed by GPP.

In addition to outcome measures, relevant medical data (type of cancer, time since diagnosis, type of treatment, and recurrence) and social demographics (age, sex, level of education, income, living situation, comorbidities, and BMI), as well as intention toward PA, healthy diet, reduced alcohol consumption, and smoking cessation will be assessed at baseline. Items assessing recurrence, use of cointerventions, BMI, and lifestyle behavioral intentions will be repeated at the 6-month measurement and 12-month assessments.

In addition, the process evaluation will be measured by (1) dose delivered: the number of intended modules accessed; (2) dose received: the extent to which participants actively engage with the material in the module; (3) satisfaction with the program; (4) practice-patient interaction: perceptions of the level of collaboration, satisfaction, and active engagement with the blended care program; and (5) context: aspects of the environment that influenced program implementation, impact, or outcomes. In addition to self-report measurements, log data from the online CAG (use of the website and individual CAG modules) will be automatically collected throughout the intervention.

Process evaluation will also take place with GPs and PNs. During the interview session at the end of the trial, they will be asked to report on (1) their satisfaction with the blended care program; (2) the type of patients involved; (3) the number of consultations carried out by the GP or PN; (4) the practice-patient interaction: perceptions of the level of cooperation, satisfaction, and active engagement with the program; and (5) the context: aspects of the environment that influenced the implementation/impact or outcomes of the program.

#### Sample Size

The effect evaluation of the online CAG intervention showed small to medium effect sizes (ES=0.20-0.40) on lifestyle behaviors at 6 months. In the current design, we expect an ES of 0.40 at 6 months follow-up [8]. Based on previous research on computer-tailored lifestyle interventions, we estimate an ES of 0.30 at 12 months [8,43-48]. Based on CAG data and other research in the primary care setting [49], we estimate an intracluster correlation of 0.03 to account for the multilevel design. Sample size calculation (ES=0.30; power=0.80; intracluster correlation=0.03, design effect 1.27) indicates that 282 participants are required for the effect study (141 in each condition). From 2 previous RCTs of online lifestyle interventions in patients or survivors of cancer, we know that dropout is 20%-25% [8,48]. Accounting for a 25% dropout, 376 survivors of cancer need to start in the RCT (188 per condition).

Furthermore, based on cancer prevalence figures, cancer survival rates, and the number of GPCs in the Netherlands, we expect that a standard GPC (consisting of 1-4 GPs and additional PNs) will have at least 20-40 survivors of cancer who meet the inclusion criteria. Therefore, to achieve a sufficient sample size, we aim to recruit approximately 40 GPCs, each of which will recruit 10 participants.

#### Randomization

A cluster-randomized design is used, which means that randomization to either the intervention or control condition takes place at the GPC level, and that all participants in a GPC are assigned to the same condition. Neither GPCs nor participants are blinded to their assigned condition during the trial. However, during recruitment, both GPCs and the researcher are unaware of the randomization outcome to ensure that participation is not influenced by assigned conditions. The randomization outcome will be defined by a computer-generated randomization list using simple randomization (1:1), which was generated before the start of recruitment and stored with a third person.

### Statistical Methods

#### Overview

The first 2 steps of the analysis will test for statistical differences at baseline in lifestyle behaviors, QoL, demographics, and psychosocial and medical symptoms between the 2 study groups. Blinding of the analyst will be applied for primary outcomes. Patterns and mechanisms of missing data will be explored. Thereby, special attention will be paid to dropouts. Randomization of the GPCs should account for an even distribution between the experimental and control conditions of socioeconomic factors or other confounding factors that may skew our results. However, in case conditions are unbalanced, these factors will be adjusted for in the analysis. If necessary, other statistical adjustment procedures will be applied to minimize the impact of any bias.

Multilevel linear regression analyses will be conducted to test for differences in residual change scores between the intervention and control groups on the primary and secondary outcomes at the 6-month follow-up and the 6- and 12-month follow-up combined. Intention-to-treat (ITT) analyses with multiple imputations will be applied. Participants will be nested within GPCs to account for potential interdependence between participants. Multilevel linear regression analyses with a random intercept for GPC level will be performed to account for possible interdependence in the effect analyses. The effects on the population will be assessed via CIs.

The primary outcome measures will be the residual change scores in lifestyle behaviors, but also differences in the secondary outcomes (anxiety and depression, fatigue, [health-related] QoL, and health-related costs). Blood pressure at 6 months will be compared between the intervention and control groups. In addition, moderation analyses will be conducted to examine different potential moderators such as age, gender, type of treatment, and level of education [8-11]. Subanalyses may be performed to explore individual differences that may be attributed to the degree of participation in the intervention. Besides factors on the level of participants, factors on the level of GPCs will be considered for further analysis. Finally, process evaluation data will be analyzed and summarized descriptively.

In addition, Bayes factor analyses will complement the primary analysis of the effects of the blended CAG intervention on the primary and secondary outcomes. A Bayes factor analysis expresses the relative strength of the evidence supporting competing hypotheses. This study examines support for the hypothesis that the blended CAG intervention has greater effects than care as usual as opposed to support for the hypothesis that the conditions do not differ or that the blended CAG intervention has a smaller effect than care as usual. In addition, the strength of support for the hypothesis that the blended CAG intervention has no smaller effects than care as usual will be compared to the support for the hypothesis that the blended CAG intervention has smaller effects than the effects found in the online-only CAG intervention, as studied in previous research [8-11]. In principle, default prior distributions will be used. It will be verified that these prior distributions are sufficiently diffuse, do not overwhelm the data, and do not destabilize the analysis.

#### Economic Evaluation

To calculate QALYs, utility scores will be obtained from the EQ-5D-5L scores and multiplied by the duration of follow-up (12 months). The economic evaluation will be conducted from a societal perspective and will therefore include medical costs (as measured by the Institute for Medical Technology Assessment Medical Consumption Questionnaire at T1), productivity costs (as measured by the Institute for Medical Technology Assessment Productivity Cost Questionnaire at T1), and costs of the blended care intervention. Intervention program costs include GP and PN training time, time for 2 consultations in the GPC, costs for program updates, and user licenses divided by a conservative number of potential annual users. Costs for the development of the intervention as well as research-specific costs were excluded.

Incremental cost-effectiveness ratios and incremental cost-utility ratios will be calculated by comparing the costs and effects (probability of maintaining a healthy lifestyle and QALYs) of the usual care group with those of the intervention group. Statistical differences in nonnormally distributed costs and QALYs will be tested using bootstrapped 1-tailed *t* tests. A cost-effectiveness acceptability curve will be constructed with the bootstrapped incremental cost-effectiveness ratio to visualize the probability that the blended care intervention is cost-effective at specific willingness-to-pay thresholds. The ceiling ratio for the cost-utility of the interventions will be set at €20,000 (US $20,900.80) per QALY. This is an accepted Dutch cutoff point for the willingness to pay for each QALY gained by preventive interventions and is commonly used to evaluate this type of intervention in the Netherlands [46,50-52].

#### Data Management

Data will be collected and handled according to the Data Management Plan that has been drafted for this project on the Data Management Plan online portal [53] and approved by ZonMw (ie, the grant provider). The progress of this study will be described in an annual report to the Medical Research Ethics Committee of Zuyderland Hospital and Zuyd University of Applied Sciences (in Dutch: *medisch-ethische toetsingscommissie van Zuyderland en Zuyd Hogeschool* [METC Z]) and the grant provider. Data will be password-protected and stored on hard disks on systems equipped with power-failure backup devices and automatic backup systems. All data will be kept confidential and anonymous. Each participant will be given a unique respondent number, not linked to a name or personal details, under which the data will be stored. Only researchers working on this project will have access to the data.

#### Potential Benefits and Risks

There are no risks or adverse effects associated with the trial. The participants in the intervention group can decide for themselves if, when, and how often they use the intervention. In addition, all the participants (intervention and control) can withdraw from this study at any time. It is expected that the use of the intervention will contribute to a healthier lifestyle, improve self-management, and have a positive impact on participants’ QoL. During this study, the control group will only participate in the online self-report questionnaires and blood pressure measurements at the GPC. They will not be denied medical care and will be able to seek additional professional support if they wish to do so. Insurance was not compulsory for this trial, as assessed by the medical ethics committee.

### Ethical Considerations

This study was approved by the METC Z before patient enrollment (NL806166.096.21, version 4.0; April 25, 2023). Informed consent (Multimedia Appendix 1) will be obtained from all the participants or legal guardians for this study. All patients will be required to provide written informed consent to participate. Participation can be discontinued at the request of the participants. Participants will receive a €10,- (10,31 USD) book voucher after completing the study. Modifications to this study protocol will be communicated to the METC Z through amendments. The METC Z has revised the informed consent materials to be given to participants and adapted them to accord with the Medical Research Involving Subjects Act. The protocol was amended to version 6.0 as of April 2023. This study conforms to the Declaration of Helsinki and is registered with the ISRCTN (International Standard Randomised Controlled Trial Number; ISRCTN12451453; registration date: December 15, 2021; last edited: September 12, 2023). All data will be kept confidential and anonymous.

## Results

This study was funded in July 2020. Data collection started in August 2022 and is likely to be completed by April 2025. As of December 2024, this study is still ongoing. Data analysis is estimated to start in the spring of 2025. A total of 127 participants have been recruited across 26 GPCs in the Netherlands. The first results are expected to be published in 2026.

## Discussion

### Overview

This study protocol describes an RCT to evaluate the effects on self-reported lifestyle behavior, psychosocial well-being, (health-related) QoL, medical consumption, and productivity costs of the blended CAG intervention integrated into general practice.

### Principal Findings

#### Overview

Previous research has investigated the effectiveness of the CAG in an online-only format [8-11]. This research found effects on PA, diet, fatigue, depression, and QoL, suggesting that the CAG is an appropriate eHealth intervention to support cancer survivorship. If similar results are found in this study, this would confirm that the online-only intervention has been appropriately adapted to the blended care context, maintaining its effectiveness. It would also suggest that the CAG could be integrated into the GPC workflow to facilitate the integration of cancer aftercare into general practice, as was proposed by the Dutch College of General Practitioners [20].

#### Comparison to Prior Work

Delivering the CAG in a blended care format improves the personalization of the care provided. This may strengthen the intervention and allow it to be better tailored to the individual recipient. Moreover, the integration of eHealth in general practice to facilitate cancer aftercare may address barriers to the uptake of cancer aftercare by general practice, as previously identified by Duineveld et al [54]. These barriers consisted of time constraints and a lack of expertise in cancer survivorship. Both of these can be complemented by effective eHealth tools such as the CAG.

### Strengths and Limitations

#### Overview

This paper describes the study protocol for an RCT of the effectiveness of a blended care intervention for cancer aftercare in general practice. Conducting an RCT strengthens our interpretation of the results by controlling for confounding through randomization. In addition, the current research is very similar to the previous research evaluating the effectiveness of the online-only CAG intervention, which allows the results to be compared and the added value of blended care to be identified. The blended care format is being added to increase the reach of our intervention, specifically targeting a population that would not access an online-only intervention on their own. The blended care protocol has been developed in cocreation with survivors, GPs, and PNs to ensure the best fit with the general practice context and the needs of survivors (MJM Smits, unpublished data, 2025). However, some challenges cannot be foreseen. Conducting the RCT in a period following the global COVID-19 pandemic may place limitations on our study protocol. For example, it may be difficult to achieve our target sample size due to recruitment problems with both GPCs and survivors of cancer. In the aftermath of COVID-19, the health care sector is facing a workforce shortage, partly due to the retirement of a large proportion of the working population [55,56]. In primary care, this is leading to the closure of GPCs as owners are not being replaced by a new generation. Meanwhile, the COVID-19 pandemic has resulted in a high rate of delayed care, further increasing the demand for GPCs and leaving little room for trial participation and research activities such as patient recruitment. This resulted in a difference in the number of GPCs recruited compared to our target. We aimed to recruit 40 GPCs, but in reality, only 26 GPCs were recruited.

#### Future Directions

Future research could further investigate the implementation of eHealth for survivors of cancer in general practice or, depending on the results of this study, in different practice contexts. Emphasis should be placed on reaching survivors who are at risk of developing comorbidities or recurrence, or who have a high burden of disease that translates into medical costs or loss of productivity.

#### Dissemination of Research Findings

Research findings will be discussed with the consortium partners and published in academic journals. This study is the next step in disseminating the CAG in a practical context. However, to translate our findings to the real world, further research should be conducted to investigate how the intervention is sustained outside of the research protocol. Depending on our findings, the RCT will be followed by an implementation study in which survivors, GPs, and other stakeholders will be invited to share their views on the integration of blended CAG into regular cancer care.

#### Implications and Relevance

The number of survivors of cancer is expected to increase in the coming decades [2]. These survivors consult their GP with issues related to cancer survivorship, resulting in a high demand for general practice [19]. To support survivors of cancer in maintaining a healthy lifestyle and managing psychosocial needs, the online CAG program was developed [57]. The program was shown to be effective in promoting healthy lifestyle behaviors and reducing fatigue, depression, and anxiety [8-11]. Structurally embedding the CAG in general practice could support GPs and PNs in caring for their patients and help reach more survivors who need support after treatment. The expected ESs are small but arguably clinically relevant given the affordability, minimal associated risks, and widespread implementability of the blended CAG intervention [58,59]. The results of this study may have implications for cancer aftercare and general practice, and may also be relevant to the wider field of eHealth implementation.

### Conclusion

The research described in this study protocol will investigate the effectiveness (and cost-effectiveness) of a blended care intervention for survivors of cancer in general practice. The blended care intervention combines the use of a proven effective eHealth program (CAG) with face-to-face consultations with a GP or PN. Integrating eHealth for cancer aftercare into general practice may help GPs and PNs in caring for the growing number of survivors of cancer and may effectively improve their QoL, leading to reduced care needs in the future. The results of this study may be relevant to the field of cancer aftercare and general practice and will add to the literature on eHealth implementation.

## Appendix

Multimedia Appendix 1 Informed consent form.

Multimedia Appendix 2 Example of the Module Referral Advice (MRA).

Multimedia Appendix 3 SPIRIT checklist. SPIRIT: Standard Protocol Items: Recommendations for Interventional Trials.

## Abbreviations

CAG: Cancer Aftercare Guide
ES: effect size
GP: general practitioner
GPC: general practice center
GPP: general practice personnel
ISRCTN: International Standard Randomised Controlled Trial Number
ITT: intention-to-treat
METC Z: Medical Research Ethics Committee of Zuyderland hospital and Zuyd University of Applied Sciences (medisch-ethische toetsingscommissie van Zuyderland en Zuyd Hogeschool)
MRA: Module Referral Advice
PA: physical activity
PN: practice nurse
QALY: quality-adjusted life year
QoL: quality of life
RCT: randomized controlled trial

## Data Availability

The datasets will be available from the corresponding author upon reasonable request.
