# Supplementary material for: Blended Care Intervention for Cancer Aftercare in General Practice Centers: Protocol for a Randomized Controlled Trial
Source: JMIR Res Protoc. 2025 Feb 12;14:e64662. doi: 10.2196/64662 (PMC11888081; doi:10.2196/64662)
Supplement: Multimedia Appendix 1 [file resprot_v14i1e64662_app1.docx]

**Bijlage C: toestemmingsformulier proefpersoon**

Behorende bij het onderzoeksprogramma Herstel na kanker.

- Ik heb de informatiebrief gelezen. Ook kon ik vragen stellen. Mijn vragen zijn goed genoeg beantwoord. Ik had genoeg tijd om te beslissen of ik meedoe.
- Ik weet dat meedoen vrijwillig is. Ook weet ik dat ik op ieder moment kan beslissen om toch niet mee te doen met het onderzoek. Of om ermee te stoppen. Ik hoef dan niet te zeggen waarom ik wil stoppen.
- Ik geef de onderzoeker toestemming om informatie op te vragen bij mijn huisarts over de status van mijn behandeling en aandoeningen die deelname aan dit onderzoek zouden kunnen belemmeren.
- Ik geef de onderzoekers toestemming om mijn gegevens en lichaamsmateriaal te verzamelen en gebruiken. De onderzoekers doen dit alleen om de onderzoeksvraag van dit onderzoek te beantwoorden.
- Ik weet dat voor de controle van het onderzoek sommige mensen al mijn gegevens kunnen inzien. Die mensen staan in deze informatiebrief. Ik geef deze mensen toestemming om mijn gegevens in te zien voor deze controle.
- Ik wil meedoen aan dit onderzoek.

Mijn naam is (proefpersoon): ………………………………..

Handtekening: ……………………… Datum : __ / __ / __

-----------------------------------------------------------------------------------------------------------------

Ik verklaar dat ik deze proefpersoon volledig heb geïnformeerd over het genoemde onderzoek.

Wordt er tijdens het onderzoek informatie bekend die die de toestemming van de proefpersoon kan beïnvloeden? Dan laat ik dit op tijd weten aan deze proefpersoon.

Naam onderzoeker (of diens vertegenwoordiger):……………………………….

Handtekening:……………………… Datum: __ / __ / __

-----------------------------------------------------------------------------------------------------------------

*De proefpersoon krijgt een volledige informatiebrief mee, samen met een getekende versie van het toestemmingsformulier.*
