# Supplementary material for: Blended Care Intervention for Cancer Aftercare in General Practice Centers: Protocol for a Randomized Controlled Trial
Source: JMIR Res Protoc. 2025 Feb 12;14:e64662. doi: 10.2196/64662 (PMC11888081; doi:10.2196/64662)

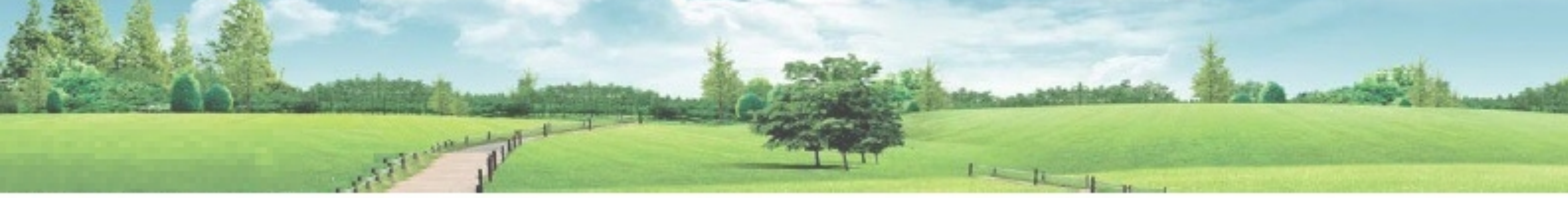

Hartelijk dank voor het invullen van de vragenlijst!

Hieronder vindt u het stoplichtenoverzicht. In dit overzicht kunt u zien hoe u op verschillende onderwerpen scoort. **Neem dit overzicht mee naar het gesprek met de huisarts of praktijkondersteuner.** U kunt het overzicht printen met de printknop rechtsboven in het scherm.

Heeft u geen printer?
Nadat u de vragenlijst heeft ingevuld krijgt u het stoplichtenoverzicht automatisch per e-mail toegestuurd naar het e-mailadres dat u heeft opgegeven. U kunt deze e-mail openen op uw smartphone of tablet als u bij de huisarts bent.
Heeft u de e-mail niet ontvangen? Controleer dan eerst uw ongewenste e-mailbox. Als u de e-mail niet kunt vinden, kunt u contact opnemen met herstelnakanker@ou.nl

Nadat u de adviezen met de huisarts of praktijkondersteuner heeft besproken kunt u aan de slag met de modules. U kunt het advies later terugvinden op uw Persoonlijke pagina.

De Kanker Nazorg Wijzer bestaat uit 9 modules: Voeding, Beweging, Roken, Alcohol, Vermoeidheid, Stemming, Werk, Relaties en Restklachten. Hieronder ziet u dat u op al deze onderwerpen een advies krijgt, afgebeeld als een ‘stoplicht’. Op de onderdelen waar de wijzer rood aanwijst liggen voor u de meeste verbeterpunten.

Stoplichtenoverzicht
De kleur van het stoplicht geeft aan wat goed gaat en waar u aan kunt gaan werken:

- **Groen:** U bent goed op weg. Het is niet noodzakelijk om deze module te volgen.
- **Oranje:** U bent al aardig op weg. Wilt u hier graag nog verder aan werken, kunt u deze module bekijken.
- **Rood:** We adviseren u om deze module te bekijken.

Wij raden u aan om de modules te volgen die gaan over de onderwerpen waar u graag hulp en/ of advies over wilt.

Tip! Kies in eerste instantie voor een module die over een onderwerp gaat waar u rood of oranje op scoort.

Voeding
Een gezonde voeding is belangrijk voor een goed herstel. Uw antwoorden wijzen erop dat u een minder gezond voedingspatroon heeft. Hier valt gezondheidswinst voor u te behalen. We adviseren u daarom om een kijkje te nemen in de module ‘Voeding’. In deze module krijgt u informatie en adviezen over gezonde voeding en hoe u de richtlijnen voor gezonde voeding het beste kunt naleven in uw dagelijks leven.

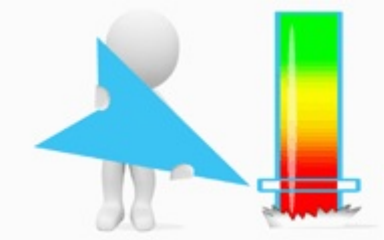

Beweging
Voldoende bewegen is belangrijk voor een goed herstel. Uw antwoorden wijzen erop dat u voldoende beweegt. De module ‘Beweging’ is voor u wellicht wat minder relevant. Wilt u toch graag wat meer informatie over hoe u om kunt gaan moet moeilijke momenten tijdens het bewegen, of wilt u een eigen beweegplan maken, dan kunt u gerust een kijkje nemen in deze module.

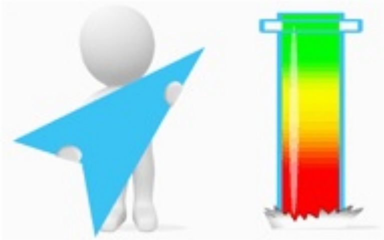

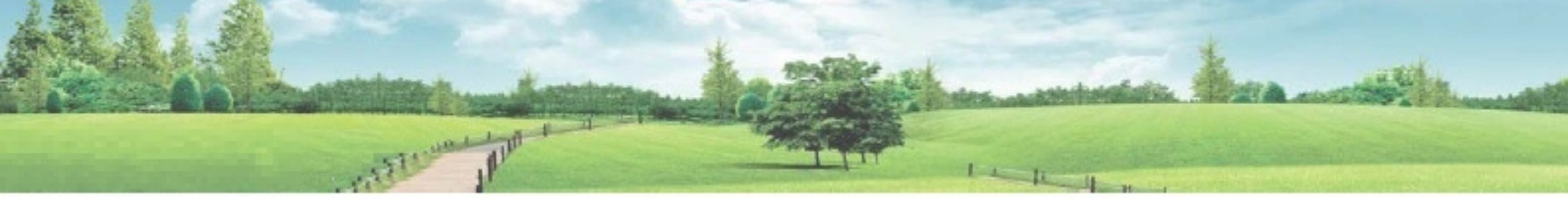

Roken
Niet roken is voor iedereen het beste, maar extra belangrijk voor mensen die kanker hebben gehad. Uit uw antwoorden blijkt dat u niet rookt. De module ‘Roken’ is voor u daarom niet relevant.

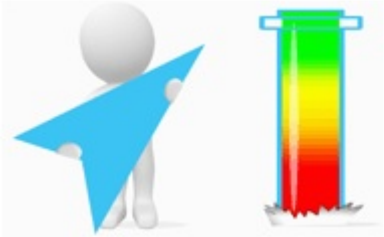

Alcohol
Uw antwoorden wijzen erop dat u gemiddeld één glas alcohol per dag drinkt. Hoewel voorheen werd gedacht dat één glas geen kwaad kon, komen we er steeds meer achter dat zelfs één glas negatieve gevolgen heeft voor de gezondheid. Voor mensen die kanker hebben gehad geldt het advies om geen alcohol te drinken. We adviseren u daarom een kijkje te nemen in de module 'Alcohol'. U krijgt hier meer informatie over alcohol en kanker en tips om te stoppen of te minderen met alcohol. Ook leert u hoe u kunt omgaan met moeilijke momenten en delen anderen hun ervaring met stoppen met alcohol in korte video’s.

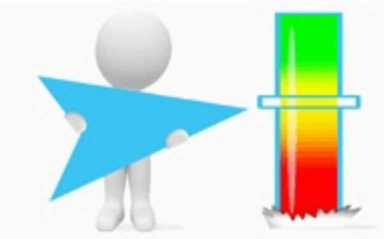

Vermoeidheid
Uw antwoorden wijzen erop dat vermoeidheid voor u een minder groot probleem is. De module ‘Vermoeidheid ’ is voor u wellicht wat minder relevant. Wilt u toch graag wat meer informatie over wat u kunt doen tegen vermoeidheid, dan kunt u gerust een kijkje nemen in deze module.

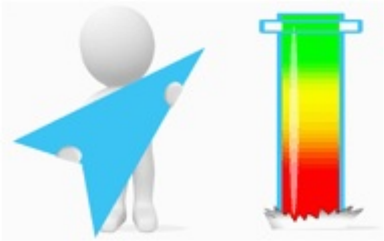

Stemming
Uw antwoorden wijzen erop dat u zich geen buitensporige zorgen maakt over uw situatie. De module ‘Stemming’ is voor u wellicht wat minder relevant. Toch kan het zijn dat u zich soms wel eens angstig of somber voelt. Neem dan gerust een keer een kijkje in deze module.

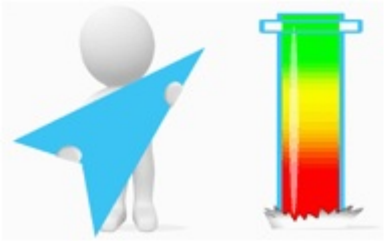

Werk
Uw antwoorden wijzen erop dat u geen duidelijke hulpvraag heeft wat betreft (terugkeer naar) werk. De module ‘Werk’ is voor u wellicht wat minder relevant. Wilt u toch graag wat meer informatie over terugkeer naar werk of over wat uw rechten en plichten zijn bij terugkeer naar werk, dan kunt u gerust een kijkje nemen in deze module.

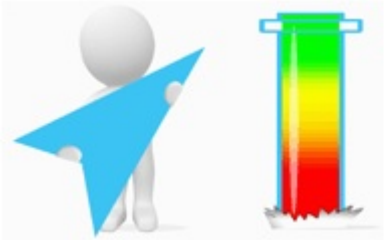

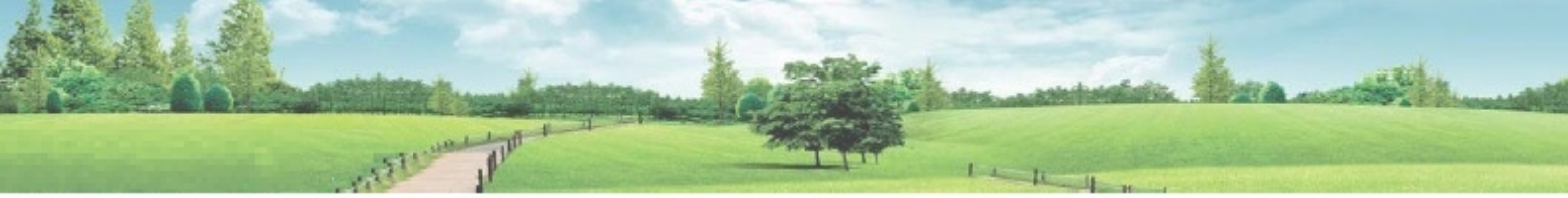

Relaties

Uw antwoorden wijzen erop dat u minder tot geen moeilijkheden ervaart met uw sociale contacten of op het gebied van intimiteit. De module ‘Relaties’ is voor u wellicht wat minder relevant. Wilt u toch graag wat meer informatie over hoe u steun kunt vragen, kanker bespreekbaar maakt, of hoe u om kunt gaan met problemen op het gebied van intimiteit en seksualiteit, dan kunt u gerust een kijkje nemen in deze module.

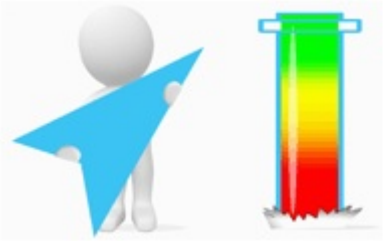

Restklachten

Wij kunnen geen persoonlijk advies geven of u de module 'Restklachten' moet bekijken. Dit komt omdat de restklachten die voormalig kankerpatiënten ervaren sterk kunnen verschillen. Wilt u meer informatie over restklachten, dan kunt u gerust een kijkje nemen in deze module.

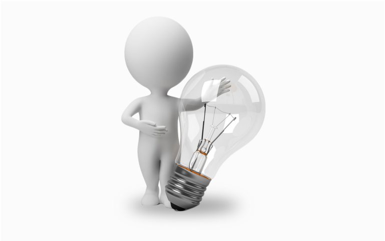

Supplement: Multimedia Appendix 2 [file resprot_v14i1e64662_app2.pdf]
